# The Effect of Discrimination on Stress among Public Housing Residents: A Comparative Study between Social-Mix and Independent Public Housing Complexes

**DOI:** 10.3390/ijerph17186788

**Published:** 2020-09-17

**Authors:** Hee-Jung Jun, Soojeong Han

**Affiliations:** 1 Department of Public Administration, Sungkyunkwan University, Seoul 03063, Korea; hjun@skku.edu; 2 Housing and Land Research Division, Korea Research Institute for Human Settlements, Sejong 30147, Korea

**Keywords:** discrimination, social exclusion, mental health, public housing, social mix, activity

## Abstract

This study aims to examine the differential effect of discrimination on stress between social-mix and independent public housing complexes. We analyzed the 2017 Seoul Public Housing Residents Panel Study data that were collected from public housing residents living in Seoul, Korea by running ordinal logit analyses. The empirical analysis shows that discrimination has a lower effect on stress in social-mix housing complexes than in independent public housing complexes. In addition, the moderating effect of community-based activities on the relationship between discrimination and stress was found in the independent public housing complex model.

## 1. Introduction

Social exclusion is a critical issue associated with public housing. Large-scale public housing concentrates poverty and can be easily distinguished from market-rate housing. With this type of environment, people can readily have prejudice and discriminate against public housing, thereby leading public housing residents to experience social stigma and isolation. Additional problems of concentrated poverty include lack of role models and economic and educational resources, which makes escaping from poverty difficult and causes antisocial activities among public housing residents [1]. Korea was not an exception to these issues. Due to the lack of residential land, public housing in Korea was built as high-rise buildings in large housing complexes and thus caused the problems of poverty concentration [2,3].

To deal with the social problems originating from concentrated poverty in large-scale public housing sites, some countries have adopted a social mix policy that mixes public housing units with market-rate housing units. An example is the HOPE VI program in the U.S. that rebuilds severely distressed public housing as socially mixed housing developments. Another example is the social mix policy in Korea that has been employed since the mid-2000s and requires newly developed housing complexes to include both market-rate and public housing units. More specifically, as of 2017, new residential development projects are undertaken by the public sector and urban redevelopment projects should include 40–50% and 10–20% of the housing units as public housing, respectively, thereby mixing households at different income levels (as far as the authors recognize, there are no particularly stringent screening criteria for living in a social-mix housing complex but those who meet the income limits based on family size and other specifications are). The rationale of adopting the social mix policy was that public housing residents living in a social-mix housing complex are less discernable from residents of market-rate housing compared to when they live in an independent public housing complex. These conditions may lower the chance of experiencing discrimination against public housing residents from the general populace and thus can reduce the negative effect of social exclusion. Additionally, antisocial activities can be lower as public housing residents learn social norms and acceptable behaviors by living and interacting with middle-to upper-income households [4,5]. Finally, urban attractiveness can be higher around social-mix housing complexes that are more likely to induce investment targeting private housing residents compared to around independent public housing complexes that concentrate poverty. 

Stress refers to internal dysfunctions resulting from external circumstances that are difficult to adjust [6]. Pearlin [7] theorizes that structural characteristics can be a stressor for individuals. Thus, the social exclusion that is caused by the structural characteristics of a large-scale public housing development can also affect stress among individual public housing residents. Although there are studies examining the health consequences of living in public housing e.g., [8,9,10], fewer studies examine the explicit relationship between discrimination and stress. More importantly, no study examines the differential effect of discrimination on stress between social-mix and independent public housing complexes. Finally, few studies analyze the effect of community-based activities that could moderate the effect of discrimination on mental health among public housing residents. In this regard, this study asks the following questions: (1) Does discrimination increase stress among public housing residents? (2) Is there a differential effect of discrimination on stress between social-mix and independent public housing complexes? (3) Do community-based activities moderate the effect of discrimination on stress?

The ecological model has been widely used in public health and emphasizes multiple levels of influences in health behaviors [11]. In the application of the ecological model, the individual and environmental levels are the most commonly defined levels. At the individual level, studies find that income, gender, age, and family type are related to mental health [11]. At the environmental level, environmental factors are usually categorized into physical and social environmental factors [12]. Studies find that physical quality of housing and neighborhoods such as the degree of dampness, mold, and pest infestation of housing, access to green/open space, transport quality, and spatial density affect mental health e.g., [13,14,15]. Those studies focusing on the social environment find that the quality of social relationships between neighbors is influential on mental health [11]. 

As Pearlin et al. [16] theorized the stress process that suggests social and economic structures that individuals experience influence mental health, many studies have examined the importance of structural aspects on mental health e.g., [17,18]. Poverty is concentrated in large-scale public housing complexes and public housing residents can be easily discriminated against and isolated; these structural aspects cause social exclusion among these residents. Along with the finding that discrimination against the disadvantaged such as racial/ethnic minorities and the poor—a transitional aspect of social exclusion—is a negative factor for mental health e.g., [19,20,21], residents living in a large-scale public housing complex are likely to be stressed by discrimination. 

Then, what about public housing residents living in a social-mix housing complex that mixes public housing with market-rate housing units? Some studies find that there are social conflicts on housing complex management between public and private housing residents [22] and public housing residents can feel social exclusion from insiders in socially mixed housing developments, but not from outsiders [23,24,25]. By contrast, those living in social-mix housing complexes may not experience discrimination as much as those living in independent public housing complexes. This is because public housing and its residents are less distinguishable and thus prejudice from outsiders can be lower for those living in social-mix housing complexes. Indeed, in Korea, Ha and Seo [2] and Suh et al. [3] find that experiencing social exclusion and discrimination was lower for those living in social-mix housing complexes than in independent public housing complexes. 

Based on the previous studies, it seems that those in both social-mix and independent public housing complexes can experience discrimination, which is a stressor. However, there are structural differences between social-mix and independent public housing complexes and thus the dynamics on the association between discrimination and stress in social-mix housing complexes are likely to be different from those in independent public housing complexes. As Joseph et al. [26] list, socially mixed communities can have such benefits as increased social networks that provide various resources for the poor, increased social control that allows an environment to have order and safety, and behavioral advantages that provide role models for the poor. Indeed, Popkin et al. [27] find a positive long-term effect—improved quality of life—for those who moved to HOPE VI housing sites from distressed public housing sites as fear of crime, which causes stress and social isolation, significantly declined. Studies also found that residential satisfaction was higher in less distressed, scattered-site public housing than large-sized, concentrated public housing [28,29,30]. In Korea’s case, Lee et al. [31] find that both public and private housing residents who used to have a negative perception on social mix began to have a more positive perception on the social mix after living in a social-mix housing complex, thereby lowering prejudice between public and private housing residents. Along with these benefits and positive outcomes of living in socially mixed housing developments, even though the residents living in social-mix housing complexes may experience discrimination from insiders, the effect of discrimination on stress may be lower in social-mix housing complexes than independent public housing complexes. In other words, given that there are various advantages to living in social-mix housing complexes, discrimination in social-mix housing complexes may not cause stress as much as is the case in independent public housing complexes. 

On the other hand, the activity theory suggests that activities are important for mental health among older people [32,33]. According to the activity theory, both physical activities (e.g., walking and exercising) and social activities (e.g., participating in social organizations) positively affect mental health by helping older people to keep busy. While few studies apply the activity theory to mental health among public housing residents, activities can also positively affect mental health among public housing residents, given that an average public housing resident is older and a relatively larger portion of public housing residents do not work compared to the general populace [34]. In addition, older people and those who do not work spend more time in their neighborhood, and thus community-based activities such as using community facilities and participating in community organizations may moderate the negative effect of discrimination on mental health among public housing residents. 

Based on the logic discussed above, we propose the following hypotheses:

**Hypothesis** **1** **(H1).** *The more public housing residents experience discrimination, the more stressed they are.*

**Hypothesis** **2** **(H2).** *Discrimination has a lower effect on stress in social-mix housing complexes than in independent public housing complexes.*

**Hypothesis** **3** **(H3).** *The effect of discrimination on stress is moderated by community-based activities.*

## 2. Materials and Methods

### 2.1. Data and Study Area

To examine the effect of discrimination on stress among public housing residents in social-mix and independent public housing complexes, this study analyzed the 2017 Seoul Public Housing Residents Panel Study data that were collected by the Seoul Housing and Communities Corporation (SH). The SH is the entity managing public housing units in the City of Seoul. Not only is Seoul the capital and largest city in Korea but also it contains a large share of public housing units (265,292 units, 18.4% of the total public housing units in 2017) [35]. 

For the panel data, the first survey was undertaken in 2016 and the 2017 survey dataset is the latest data available for this study. The 2017 survey was conducted by personal interviews on tablet PCs between September and December in 2017. The stratified random sampling process was employed to build a representative sample for the 2016 survey: First, public housing complexes were proportionally sampled in each of the four regional housing welfare centers in Seoul. Next, five households who are over 19 years old in the list of public housing residents were randomly selected from the public housing complexes to take the survey. The 2017 survey was taken to the same sample respondents from the 2016 survey and its response rate was relatively high, 91.2% [36]. Of a total sample of 5265, we excluded survey responses that were collected from public housing residents not living in a housing complex and used the 4574 responses that contain the responses from public housing residents living in a housing complex, since this study aims to compare the differential effect of discrimination on stress between social-mix housing complexes (1898) and independent public housing complexes (2676). 

The data include information on the type of public housing complex in which a resident lives (i.e., either a social-mix or independent public housing complex), physical environment characteristics about housing and the neighborhood, characteristics of the neighborhood social environment, and additional self-reported data such as whether the resident had experienced discrimination, health status, including information on the level of perceived stress and whether the resident had chronic diseases, use of community facilities, participation in community organizations, and demographic characteristics. Thus, the dataset is sufficient to test the proposed hypotheses in this study. 

### 2.2. Analytic Strategy and Variables

To test the proposed hypotheses, we first ran an ordinal logit analysis by using the pooled sample and use discrimination as the key independent variable on stress among public housing residents. Then, we ran ordinal logit analyses in each of the two subgroups, those living in social-mix and independent public housing complexes. Finally, we ran the moderating effect model that includes interaction terms between community-based activities and discrimination. 

The dependent variable in this study is the level of perceived stress (hereafter the stress level). Public housing residents were required to answer on a 4-point Likert scale (very low, low, high, and very high the following stress question: “How much stress do you usually experience in your daily life?” The key independent variable is whether the respondent experienced discrimination due to living in public housing (yes = 1, no = 0). In testing the moderating effect of community-based activities, we included the variables of whether the resident uses community facilities such as community space for the elderly and meetings and the gym (yes = 1, no = 0) and participates in community organizations such as public housing resident and elderly associations and community clubs (yes = 1, no = 0) and their interaction terms with the discrimination variable. 

In accordance with the ecological model, we also included both individual-level and environmental factors to estimate the stress level among public housing residents. For the individual-level factors, we included the following demographic characteristics: man (reference woman = 0), working (reference not working = 0), age (in years), high-school graduate (reference below high-school graduate = 0), yearly household income (10,000 Korean Won), household size (in number), years of residence (in years), and having a chronic disease (reference not having a chronic disease = 0). At the environmental level, we included physical and social environmental characteristics of housing and neighborhood that individuals assessed. For physical housing quality, we included a housing condition variable that averaged the assessed physical quality of three indoor housing environments: moisture (mold, water leaks, and flood control), finishing (paint and flooring conditions), and systems (the cooling and heating system and electric lighting conditions). For the physical environmental quality of the neighborhood, we included variables on the satisfaction levels with green space and recreational space, safety from crime, access to public transportation, educational environment, and access to neighborhood facilities (the composite measure by summing and averaging the satisfaction levels with access to retail stores, public institutions, cultural facilities, and hospitals). Those variables were measured as ordinal variables, with ranges from 1 to 4 (very bad to very good). For the social-environmental quality of the neighborhood, we included the composite measure of social capital (the mean of network, reciprocity, and trust with neighbors as Putnam [37] suggested). The levels of network, reciprocity, and trust with neighbors were measured with the questions of “How close are you to your neighbors?”, “Are you willing to help in case your neighbors need help” and “Do you think you can trust your neighbors?”, respectively, and these measures range from 1 to 4 (very low to very high). 

### 2.3. Descriptive Statistics

Table 1 reports the descriptive statistics. The stress level—the dependent variable—of the pooled sample is 2.78, which is between low and high levels of stress and the t-test analysis shows there is no statistically significant difference in the stress level between social-mix and independent public housing complex groups. The mean value of discrimination, the key independent variable, is 0.06, which suggests 6% of the sample public housing residents experienced discrimination due to living in public housing. Although the share of having discrimination is higher in the independent public housing complex group (0.06) than the social-mix housing complex group (0.05), the difference between the two groups is not statistically significant, unexpectedly. 

The individual-level factors describe the average characteristics of public housing residents. For the pooled sample, there are more men (66%) than women and people working than not working. The mean age (61.82) suggests that an average public housing resident is older. Also, the share of high-school graduates (26%) and yearly household income (about 59% of the median household income in Korea in 2017) shows the low socio-economic status of an average public housing resident. The average household size and years of residence are close to 3 people per household and having lived there for more than 10 years, respectively. Finally, a relatively large share (41%) of public housing residents has a chronic disease. In comparison between social-mix and independent public housing complexes, public housing residents in the subgroups are statistically significantly different from each other. There are more men, people working, younger people, high-school graduates, and higher-income people in the social-mix housing complex group compared to the independent public housing complex group. The average household size is also larger and the length of residence is shorter in the social-mix housing complex group compared to the independent public housing complex group. Finally, the share of people with a chronic disease is smaller for those living in social-mix housing complexes than independent public housing complexes. The comparisons of individual-level factors between the subgroups suggest that, overall, socio-economic status is higher for the social-mix housing complex group than the independent public housing complex group.

The descriptive statistics of the physical environmental factors show that the average public housing resident is, overall, satisfied with their housing and neighborhood as the mean values of assessed housing conditions and satisfaction levels with park and recreational facilities, safety from crime, access to public transit, educational environment, and access to neighborhood facilities are between satisfied (3) and very satisfied (4). Compared to the higher levels of satisfaction with physical environmental factors on housing and neighborhood, the assessment with the neighborhood social environment is relatively lower as the level of social capital is 2.84 out of 4. Twenty one percent of the respondents reported that they use community facilities and only four percentage of the respondents reported that they participate in a community organization. In comparison between the subgroups, the assessed quality and satisfaction levels of housing conditions, access to public transit, educational environment, and access to neighborhood facilities are higher in the independent public housing complex group than the social-mix housing complex group. By contrast, the satisfaction levels with green and recreational areas and safety from crime, the assessed level of social capital, and the shares of people using community facilities and participating in a community organization are all higher in the social-mix housing complex group than the independent public housing complex group. 

## 3. Results

Table 2 shows the ordinal logit estimates in the pooled model, including responses from public housing residents living both in social-mix and independent public housing complexes. As hypothesized, discrimination is positively related to stress among public housing residents. Given that the odds ratio of the discrimination variable is 1.76, those who experience discrimination due to living in public housing are 76% more likely to be stressed than those who do not are. 

To examine if there is a differential effect of discrimination on stress among public housing residents between the two groups, we ran another ordinal logit analysis by including the social-mix housing complex (dummy) variable and its interaction term with the discrimination variable in the pooled model. As shown in Table 2, the interaction term is negatively related to stress among public housing residents. Given that the discrimination variable is positively related to stress, the negative interaction term suggests that living in a social-mix housing complex reduces the effect of discrimination on stress. In other words, even though discrimination can increase stress among public housing residents, increased stress is lower for those living in a social-mix housing complex. This finding suggests that the social-mix policy is successful in reducing the stress associated with discrimination against public housing residents and thus supports the second hypothesis that discrimination has a lower effect on stress in social-mix housing complexes than independent public housing complexes.

On the other hand, the social-mix housing complex variable is positively related to the stress level. That is, those living in social-mix housing complexes report higher levels of stress than those living in independent public housing complexes, holding constant others. It seems that there are alternative factors increasing stress among public housing residents living in social-mix housing complexes. 

The relationships between individual-level and environmental factors and stress levels are mostly as expected. The variables of age, high-school graduate, housing condition, safety from crime, educational environment, and social capital were negatively related to stress (we examined if age has a non-linear relationship with stress by including the squared term of age. As the squared term of age was not statistically significant in all models, we dropped the variable from the final models). Conversely, the variables of working, household size, years of residence, chronic disease, and access to public transit were positively related to stress. While the directions of other variables make sense, one might wonder why years of residence is positively related to stress. This finding is consistent with Han and Jun’s findings [38]. Their study finds that the level of depression was higher for the comparison group—those who lived in public housing for seven years than the control group—those who lived in public housing for three years. This may be because those who lived in public housing longer are more stressed as they cannot escape from poverty. In addition, the positive effect of access to public transit on stress is unexpected. This may be because of the spatial characteristics of Seoul where access to public transit is high where densities are very high. Given that studies find that high density is more likely to increase stress e.g., [39], the unexpected relationship may be because of the city’s complex environment with higher densities that can cause stress.

As shown in Table 2, the effect of discrimination is lowered by living in social-mix housing complexes. To further explore which factors are associated with stress among public housing residents in subgroups, we subdivided the pooled sample and ran ordinal logit analyses in the social-mix and independent public housing complex groups. As shown in Table 3, the most critical difference between the subgroup model estimates is that the discrimination variable is statistically insignificant in the social-mix housing complex model while it is statistically significant with a positive sign in the independent public housing complex model as in the pooled model. That is, discrimination increases stress among those living in independent public housing complexes while it does not have a statistically significant impact on stress among those living in social-mix housing complexes. Additionally, the odds ratio (2.11) of the discrimination variable in the independent public housing complex model indicates that those who experience discrimination due to living public housing are 111% more likely to be stressed than those who do not are. 

Then, why is discrimination influential on stress only in independent public housing complexes? In the descriptive statistics, there was no statistically significant difference in the stress level and discrimination between social-mix and independent public housing complexes. However, not only is the effect of discrimination reduced by the interaction between discrimination and social-mix housing complex in the pooled model but also discrimination is positively related to stress only in the independent public housing complex model, not in the social-mix housing complex model. 

The insignificance of the discrimination variable in the social-mix housing complex model may be explained by two possibilities. First, it may suggest that the social mix policy has addressed the problem of social exclusion and, at least, removed the discrimination effect on stress among public housing residents. Given that there is no statistically significant difference in the discrimination variable in the chi-square test, it seems that the types or levels of discrimination in social-mix housing complexes do not increase the stress level among public housing residents. While the survey data do not provide information about specific levels of discrimination, the level of discrimination may be low enough that it does not reach some critical point that increases the stress level among public housing residents living in social-mix housing complexes. In other words, along with various benefits and positive outcomes of living in social-mix housing complexes (e.g., increased social networks, living in a more attractive urban environment, and reduced prejudice between public and private housing residents) discussed above, the type of discrimination in social-mix housing complexes may not increase stress among public housing residents as much as in independent public housing complexes. 

Second, it seems that those living in social-mix housing complexes experience stress more because of other factors, not discrimination. As shown in Table 3, as compared to the independent public housing complex model where the working and educational environment variables are not statistically significant, the working and educational environment variables are positively and negatively related to stress among public housing residents, respectively, in the social-mix housing complex model. Due to the structural differences in addressing social exclusion between the two different types of public housing complexes, it seems that public housing residents living in social-mix housing complexes are not stressed by discrimination but stressed when they work and assess their educational environment as being worse. 

While other variables are similarly related to stress among public housing residents as in the pooled model, housing conditions turned out to be statistically insignificant in the social-mix housing complex model. This may be because public housing units in social-mix housing complexes are relatively newer than those in independent public housing complexes and thus the physical quality of housing is not influential on stress among those living in social-mix housing complexes. Finally, household size, which was statistically significant with a positive sign in the pooled model, became statistically insignificant in both models. It seems that the marginal number of samples affecting stress was included in the pooled model, which became insignificant when divided into the subgroups. 

Table 4 reports the moderating effect models that include community-based activities variables and their interaction terms with discrimination. As shown in the table, using community facilities and participating in community organizations present differential effects between the two models. While the two community-based activities variables are statistically significant with a negative sign in the social-mix housing complex model, the participating in community organizations variable is only statistically significant with a negative sign in the pooled model. In the independent public housing complex model, none of the community-based activities variables is statistically significant. That is, the results suggest that community-based activities can reduce stress among public housing residents in social-mix housing complexes, not in independent public housing complexes. 

On the other hand, the interaction term between discrimination and using community facilities is statistically significant with a negative sign only in the independent public housing complexes model. This result implies that, as proposed by the third hypothesis, the effect of discrimination on stress is moderated by using community facilities, although community-based activities do not directly reduce stress among public housing residents in independent public housing complexes. In other words, those who experience discrimination due to living in public housing are stressed but the effect from discrimination is lower when using community facilities and this moderating effect only applies to the independent public housing complex model that presented the statistically significant effect of discrimination on stress. 

## 4. Conclusions

Social exclusion is a major problem in public housing and can have a critical impact on residents’ mental health. To our knowledge, this is the first study comparing the differential effect of discrimination on stress among public housing residents between social-mix and independent public housing complexes. To fill the gap in the literature, we examined the effect of discrimination on stress among public housing residents in the pooled model and subgroup models, including the social-mix and independent public housing complex groups. We also examined if community-based activities can moderate the effect of discrimination on stress among public housing residents.

The first hypothesis, “The more public housing residents experience discrimination, the more stressed they are”, was supported. In the pooled model, the discrimination variable was statistically significant with a positive sign. That is, discrimination experience due to living in public housing negatively affects mental health among public housing residents. 

The second hypothesis, “Discrimination has a lower effect on stress in social-mix housing complexes than in independent public housing complexes”, was also supported. The interaction term between the discrimination and social-mix housing complex variables was negatively related to stress in the pooled model, which means that living in social-mix housing complexes reduces the effect of discrimination. When the pooled model was subdivided, discrimination was statistically insignificant in the social-mix housing complex model, but positively related to stress in the independent public housing complex model. The differential effect of discrimination between the subgroups suggests that the social mix policy was successful in addressing social exclusion and removing the effect of discrimination on stress among public housing residents. 

The third hypothesis, “The effect of discrimination on stress is moderated by community-based activities”, was partially supported. Although none of the community-based activities variables was statistically significant, we found that the interaction term between using community facilities and discrimination is statistically significant with a negative sign in the independent public housing complex model. That is, although the community-based activities do not directly reduce stress among public housing residents, the effect of discrimination is moderated when using community facilities for those living in independent public housing complexes. This finding suggests the importance of community-based activities in reducing stress among public housing residents living in independent public housing complexes that are increased by discrimination.

These results suggest that policymakers, and housing and public health practitioners should acknowledge that minimizing discrimination should be the first priority in managing stress among the general population of public housing residents. Given finding the effectiveness of the social mix policy in reducing the effect of discrimination on stress, housing policy should continue its direction to develop socially mixed communities. Fortunately, the Korea government announced in 2020 to expand the social-mix policy by increasing the required ratio of public housing in newly developed housing complexes [40]. Those countries where social exclusion against public housing residents is a serious social issue may want to take a similar approach to Korea for social harmony. Finally, given that there is still a large portion of independent public housing complexes in Korea as well as many countries, policymakers should acknowledge that the effect of discrimination on stress for those living in independent public housing complexes can be moderated upon more involved in community-based activities. Kim and Sung [41] point that, despite enough space for community-based activities in public housing complexes, community facilities are not well managed and community programs do not meet the needs of the residents. Thus, there should be policy efforts to improve the quality of community facilities and encourage community participation by providing community programs that meet the needs of public housing residents as a strategy for managing stress from discrimination.

This study contributes to the literature by examining the differential effect of discrimination on stress among public housing residents between social-mix and independent public housing complexes. Nevertheless, there are still limitations to this study. Because the data do not provide information about specific levels of discrimination but provide information only on whether the respondent experienced discrimination, we could not analyze how different magnitudes of discrimination affect stress among public housing residents. Like the discrimination variable, community-based activities variables only measured whether the respondents were involved in community-based activities rather than the specific levels of involvement in community-based activities. Future studies that employ more affluent data on these variables will be able to analyze more specific effects of those variables on stress among public housing residents and their differential effects on stress between those living in social-mix and independent public housing complexes.

## Figures and Tables

**Table 1:** Descriptive statistics.

Variables	Pooled				Social-Mix Housing Complex				Independent Public Housing Complex				Difference (Mean/%)
	Mean	S.D.	Min	Max	Mean	S.D.	Min	Max	Mean	S.D.	Min	Max	
Stress level	2.78	0.61	1	4	2.80	0.61	1	4	2.76	0.62	1	4	0.03
Discrimination (ref. no discrimination)	0.06	-	0	1	0.05	-	0	1	0.06	-	0	1	−0.01
Man (ref. woman)	0.66	-	0	1	0.73	-	0	1	0.62	-	0	1	0.01 ***
Working (ref. Not working)	0.61	-	0	1	0.70	-	0	1	0.54	-	0	1	0.15 ***
Age	61.82	13.46	24	101	59.6	13.66	24	99	63.3	13.17	24	101	−3.66 ***
High-school graduate (ref. below high-school)	0.26	-	0	1	0.35	-	0	1	0.19	-	0	1	0.15 ***
Yearly household income (10,000 Won)	2577.5	1966.0	0	15,000	3236.1	1964.7	0	15,000	2571.3	1753.9	0	11,050	664.8 ***
Household size	2.85	1.28	1	8	3.15	1.28	1	8	2.63	1.24	1	8	0.51 ***
Years of residence	10.40	7.48	0	28	7.13	5.65	0	26	12.76	7.73	0	28	−5.62 ***
Chronic disease (ref. no chronic disease)	0.41	-	0	1	0.33	-	0	1	0.46	-	0	1	−0.12 ***
Housing condition	3.15	0.65	1	4	3.12	0.68	1	4	3.17	0.63	1	4	−0.04 **
Green/recreation area	3.30	0.62	1	4	3.33	0.61	1	4	3.27	0.61	1	4	0.06 ***
Safety from crime	3.28	0.63	1	4	3.34	0.62	1	4	3.25	0.63	1	4	0.09 ***
Access to public transit	3.25	0.68	1	4	3.17	0.71	1	4	3.31	0.64	1	4	−0.13 ***
Educational environment	3.12	0.66	1	4	3.04	0.69	1	4	3.18	0.64	1	4	−0.13 ***
Access to neighborhood facilities	3.07	0.60	1	4	2.95	0.61	1	4	3.16	0.57	1	4	−0.21 ***
Social capital	2.84	0.43	1	4	2.87	0.41	1	4	2.81	0.45	1	4	0.05 ***
Using community facilities (ref. not using community facilities)	0.21	-	0	1	0.26	-	0	1	0.17	-	0	1	0.09 ***
Community participation (ref. no participation)	0.04	-	0	1	0.06	-	0	1	0.03	-	0	1	0.03 ***
N	4574				1898				2676				-

*** *p* < 0.01, ** *p* < 0.05.

**Table 2:** Stress level estimates: the pooled model and the pooled model with the interaction term between discrimination and social-mix housing complex.

Variables	Pooled Model		Pooled Model with Interaction Term of Social-Mix Housing Complex Var.	
	Beta (S.E.)	Odds Ratio	Beta (S.E.)	Odds Ratio
*Discrimination* (ref. no discrimination)	0.06 *** (0.03)	1.75	0.08 *** (0.04)	2.16
*Social mix housing complex* (ref. independent public housing complex)	-	-	0.05 *** (0.02)	1.29
Discrimination *Social- mix housing complex	-	-	−0.03 * (0.07)	0.57
Man (ref. woman)	−0.00 (0.20)	0.98	−0.00 (0.02)	0.97
Working (ref. not working)	0.04 ** (0.02)	1.16	0.03 ** (0.02)	1.14
Age	−0.09 *** (0.00)	0.98	−0.09 *** (0.00)	0.98
High-school graduate (ref. below high-school)	−0.06 *** (0.02)	0.74	−0.05 *** (0.02)	0.73
Yearly household income	0.03 (6.31)	1.00	0.02 (6.31)	1.00
Household size	0.04 * (0.00)	1.06	0.03 * (0.00)	1.06
Years of residence	0.03 * (0.00)	1.00	0.04 *** (0.00)	1.01
Chronic disease (ref. no chronic disease)	0.05 *** (0.02)	1.21	0.04 *** (0.02)	1.22
Housing condition	−0.05 *** (0.01)	0.84	−0.04 *** (0.01)	0.86
Green/recreation area	−0.00 (0.01)	0.97	−0.00 (0.01)	0.96
Safety from crime	−0.09 *** (0.01)	0.72	−0.09 *** (0.01)	0.71
Access to public transit	0.07 *** (0.01)	1.24	0.06 *** (0.01)	1.24
Educational environment	−0.05 ** (0.01)	0.83	−0.04 ** (0.01)	0.84
Access to neighborhood facilities	−0.02 (0.02)	0.95	−0.01 (0.02)	0.97
Social capital	−0.18 *** (0.02)	0.39	−0.18 *** (0.02)	0.38
Number of observations	4547		4547	
Log likelihood	−4042.09		−4034.44	
*X*^2^ value	405.92 ***		421.23 ***	
Pseudo *R*^2^	0.05		0.04	

*** *p* < 0.01, ** *p* < 0.05, * *p* < 0.1.

**Table 3:** Stress level estimates: the subgroup models of social-mix and independent public housing complexes.

Variables	Social-Mix Public Housing Complex		Independent Public Housing Complex	
	Beta (S.E.)	Odds Ratio	Beta (S.E.)	Odds Ratio
*Discrimination*	0.02 (0.05)	1.23	0.08 *** (0.04)	2.11
Man (ref. woman)	0.01 (0.03)	1.03	−0.01 (0.02)	0.93
Working (ref. not working)	0.08 *** (0.03)	1.40	0.01 (0.02)	1.02
Age	−0.11 *** (0.00)	0.98	−0.07 *** (0.00)	0.98
High-school graduate (ref. below high-school)	−0.06 ** (0.03)	0.71	−0.05 *** (0.03)	0.74
Yearly household income	0.02 (8.92)	1.00	0.03 (8.97)	1.00
Household size	0.04 (0.01)	1.06	0.04 (0.01)	1.07
Years of residence	0.06 ** (0.00)	1.02	0.04 * (0.00)	1.01
Chronic disease (ref. no chronic disease)	0.05 * (0.03)	1.21	0.04 ** (0.02)	1.20
Housing condition	−0.03 (0.02)	0.92	−0.06 ** (0.02)	0.81
Green/recreation area	0.01 (0.03)	1.04	−0.02 (0.02)	0.90
Safety from crime	−0.08 ** (0.03)	0.74	−0.11 *** (0.02)	0.68
Access to public transit	0.08 *** (0.02)	1.29	0.05 ** (0.02)	1.19
Educational environment	−0.11 *** (0.02)	0.68	0.02 (0.02)	1.06
Access to neighborhood facilities	0.01 (0.02)	1.06	−0.04 (0.03)	0.89
Social capital	−0.13 *** (0.03)	0.51	−0.22 *** (0.02)	0.32
Number of observations	1883		2664	
Log-likelihood	−1665.89		−2349.66	
*X*^2^ value	139.27 ***		315.84 ***	
Pseudo *R*^2^ Pseudo *R*^2^	0.04		0.06	

*** *p* < 0.01, ** *p* < 0.05, * *p* < 0.1.

**Table 4:** Stress level estimates: the moderating effect of community-based activities.

Variables	Pooled		Social-Mix Public Housing Complex		Independent Public Housing Complex	
	Beta (S.E.)	Odds Ratio	Beta (S.E.)	Odds Ratio	Beta (S.E.)	Odds Ratio
*Discrimination*	0.07 *** (0.04)	1.97	0.02 (0.07)	1.15	0.10 *** (0.05)	2.59
Discrimination * Using community facilities	−0.02 (0.08)	0.66	−0.02 (0.14)	1.40	−0.05 ** (0.11)	0.40
Discrimination * Community participation	0.00 (0.12)	1.05	0.01 (0.15)	1.16	0.00 (0.20)	0.95
*Using community facilities*	−0.02 (0.02)	0.90	−0.08 *** (0.03)	0.69	0.01 (0.03)	1.05
*Community participation*	−0.03 * (0.04)	0.74	−0.05 * (0.06)	0.67	−0.03 (0.06)	0.71
Man (ref. woman)	−0.00 (0.02)	0.98	0.01 (0.03)	1.03	−0.01 (0.02)	0.95
Working (ref. not working)	0.04 ** (0.02)	1.16	0.08 *** (0.03)	1.38	0.01 (0.02)	1.02
Age	−0.08 *** (0.00)	0.98	−0.12 *** (0.00)	0.98	−0.07 *** (0.00)	0.98
High-school graduate (ref. below high-school)	−0.05 *** (0.02)	0.76	−0.06 ** (0.03)	0.74	−0.05 *** (0.03)	0.74
Yearly household income	0.02 (6.31)	1.00	0.02 (8.91)	1.00	0.03 (8.97)	1.00
Household size	0.03 (0.00)	1.06	0.04 (0.01)	1.07	0.04 (0.01)	1.06
Years of residence	0.02 * (0.00)	1.00	0.05 ** (0.00)	1.02	0.04 * (0.00)	1.01
Chronic disease (ref. no chronic disease)	0.05 *** (0.02)	1.22	0.05 ** (0.03)	1.24	0.05 ** (0.02)	1.21
Housing condition	−0.05 *** (0.01)	0.83	−0.03 (0.02)	0.92	−0.06 ** (0.02)	0.81
Green/recreation area	−0.00 (0.01)	0.98	0.02 (0.03)	1.07	−0.02 (0.02)	0.89
Safety from crime	−0.08 *** (0.02)	0.73	−0.08 ** (0.03)	0.75	−0.11 *** (0.02)	0.68
Access to public transit	0.06 *** (0.01)	1.23	0.08 *** (0.02)	1.27	0.05 ** (0.02)	1.19
Educational environment	−0.04 ** (0.01)	0.84	−0.11 *** (0.02)	0.69	0.02 (0.02)	1.07
Access to neighborhood facilities	−0.02 (0.02)	0.95	0.00 (0.02)	1.03	−0.05 (0.03)	0.88
Social capital	−0.17 *** (0.02)	0.40	−0.11 *** (0.03)	0.56	−0.22 *** (0.02)	0.32
Number of observations	4547		1883		2664	
Log likelihood	−4036.74		−1657.59		−2345.78	
*X*^2^ value	416.62 ***		155.87 ***		323.59 ***	
Pseudo *R*^2^	0.04		0.04		0.06	

*** *p* < 0.01, ** *p* < 0.05, * *p* < 0.1.
